# Gene-Sex Interaction in Non-Syndromic Orofacial Cleft Subtypes: A Case-Control Study Among the Vietnamese Population

**DOI:** 10.3390/genes16080853

**Published:** 2025-07-22

**Authors:** Le Kha Anh, Teruyuki Niimi, Satoshi Suzuki, Toko Hayakawa, Ken Kitagawa, Chisato Sakuma, Hideto Imura, Hisataka Kondo, Nguyen Huu Tu, Tong Minh Son, Vo Truong Nhu Ngoc, Tran Phuong Thao, Nguyen Minh Duc, Pham Nguyen Gia Loc, Hiroo Furukawa, Nagana Natsume, Nagato Natsume

**Affiliations:** 1 Division of Research and Treatment for Oral Maxillofacial Congenital Anomalies, Aichi Gakuin University, Nagoya 464-8651, Japan; lekhaanh268@gmail.com (L.K.A.); niimi@dpc.agu.ac.jp (T.N.); zsatoshi@hotmail.com (S.S.); 9646ken@gmail.com (K.K.); char_nyoko@yahoo.co.jp (C.S.); h-imura@dpc.agu.ac.jp (H.I.); thanhan311099@gmail.com (T.P.T.); minhduc.dentist@gmail.com (N.M.D.); gialocp@gmail.com (P.N.G.L.); ag223d15@dpc.agu.ac.jp (N.N.); 2 School of Dentistry, Hanoi Medical University, Hanoi 10000, Vietnam; tongminhson@hmu.edu.vn (T.M.S.); nhungoc@hmu.edu.vn (V.T.N.N.); 3 Cleft Lip and Plate Center, Aichi Gakuin University Dental Hospital, Nagoya 464-8651, Japan; hayakawa@dpc.agu.ac.jp; 4 Division of Speech, Hearing, and Language, Aichi Gakuin University Dental Hospital, Nagoya 464-8651, Japan; hfuru@dpc.agu.ac.jp; 5 Junior College, Aichi Gakuin University, Nagoya 464-0037, Japan; kondoh@dpc.agu.ac.jp; 6 Department of Anesthesia-Resuscitation, Hanoi Medical University, Hanoi 10000, Vietnam; nguyenhuutu@hmu.edu.vn; 7 Odonto-Maxillo Facial Hospital of Ho Chi Minh City, Ho Chi Minh City 71000, Vietnam

**Keywords:** *WNT3*, *NOG*, non-syndromic orofacial clefts, Vietnamese

## Abstract

Background: Non-syndromic orofacial clefts (NSOFCs) are one of the common congenital malformations in Vietnam, with 1.4 per 1000 live births, with notable sex differences in occurrence. This case–control study aims to investigate potential sex-specific interactions of *WNT3* and *NOG* polymorphisms across NSOFC subtypes in a Vietnamese population. Methods: A total of 720 participants were separated into 4 groups with a male/female ratio of 1:1 (160 individuals with cleft lip and palate (NSCLP), 160 with cleft lip only (NSCLO), 160 with cleft palate only (NSCPO), 240 healthy controls). Two single-nucleotide polymorphisms (SNPs), rs3809857 and rs227731, were genotyped by using the StepOnePlus Real-Time PCR System. Results: The most significant findings were found in the male NSCLO group under a recessive model of *WNT3* rs3809857 after applying Bonferroni correction, as a five-fold protective factor with OR = 0.18 (95% confidence interval: 0.05–0.64, *p* = 0.0033). Additionally, the weak or moderate protective association between rs3809857 and male NSCLP was found with *p* < 0.05 under the dominant model. However, there were no significant findings in the female NSOFC subtypes associated with *WNT3*. Conversely, *NOG* rs227731 results showed a weak increased risk in female NSCLO and NSCPO with *p* < 0.05. Conclusion: this study identified the critical role of *WNT3* rs3809857 in reducing NSCLO risk in males. These findings support the potential influence of sex as a modifying factor in the genetic susceptibility to non-syndromic orofacial clefts.

## 1. Introduction

Non-syndromic orofacial clefts (NSOFCs) represent one of the most common congenital craniofacial anomalies worldwide [1]. Approximately 70% of all orofacial clefts (OFCs) are considered non-syndromic, a complex and multifactorial condition arising from the interaction of multiple genetic predispositions and environmental influences [2]. These malformations are typically classified into three major groups based on the affected anatomical structures: non-syndromic cleft palate only (NSCPO), non-syndromic cleft lip only (NSCLO), and non-syndromic cleft lip and palate (NSCLP). The global incidence of NSOFCs is estimated to be approximately 1 in 700 live births, with notable variation across geographic regions and ethnic populations [2]. The highest prevalence rates have been observed in Asian populations, particularly in Vietnam (1.4 per 1000 live births) [1,3]. The impact extends beyond aesthetics, affecting crucial functions such as feeding, speech, hearing, and respiration, and can lead to social adjustment challenges and reduce the quality of life [4].

Sex-related differences are also well documented in the epidemiology of NSOFCs. Males are approximately twice as likely to present with cleft lip (CL) or cleft lip with cleft palate (CLP) compared to females, whereas females are about twice as likely to have cleft palate only (CP) [5]. These sex-based disparities are believed to stem from differences in gene expression levels and regulatory patterns. Genome-wide studies have supported this, identifying sex-dependent genetic interactions, such as a novel risk locus at chromosome 8p22 and a sex-specific interaction at the intergenic 10q21 locus, both associated with altered NSCL/P risk in males and females [6,7]. In the Vietnamese population, research from our laboratory has identified sex-specific genetic interactions, with *MEOX2* showing a significant association with gender [8].

The wingless-type MMTV integration site (WNT) signaling pathway plays an essential role in embryonic development, including craniofacial morphogenesis [9,10,11]. At the molecular level, facial formation is regulated by the *PBX-WNT-TP63-IRF6* signaling cascade, which facilitates epithelial apoptosis necessary for proper facial fusion [12,13]. A key component of the WNT family, *WNT3* has been repeatedly implicated in genetic studies as being associated with NSOFCs in humans, and similar associations have been confirmed in mouse models [14,15].

The *NOG* gene, encoding noggin, is a known antagonist of bone morphogenetic protein (BMP) signaling, a critical pathway in embryogenesis and facial structure formation [16]. Genome-wide association studies (GWAS) have suggested a link between rs227731 in *NOG* and the risk of NSOFCs [17]. Moreover, a recent meta-analysis has indicated that this polymorphism may increase the risk of NSCL/P specifically in Caucasian populations [18].

The present study conducted a case–control design to investigate the potential sex interactions with *WNT3* rs3809857 and *NOG* rs227731 across various NSOFC subtypes in a Vietnamese population.

## 2. Materials and Methods

### 2.1. Subject Recruitment

A case–control study was conducted involving a total of 720 participants, recruited from the Odonto Maxillofacial Hospital in Ho Chi Minh City, Vietnam. The case group consisted of three subtypes of NSOFC: 160 individuals with NSCLO, 160 with NSCLP, and 160 with NSCPO. The control group comprised 240 healthy individuals with no clinical evidence or family history of NSOFCs or other congenital malformations. All four participant groups were selected with a male/female ratio of 1:1 in each group. This balanced design, a form of stratified sampling, enhances the statistical power and precision of our research, particularly for dissecting the distinct genetic underpinnings of these subtypes, including NSCLP, NSCLO, and NSCPO, to investigate etiological heterogeneity within the complex trait of orofacial cleft. While this design enhances the power for subtype-specific analysis, it creates a sample that is not representative of the general population’s cleft prevalence. Therefore, we decided to compare every single NSOFC subtype with control and refrained from conducting analyses on the pooled NSOFC and NSCL/P groups to ensure the validity of our conclusions.

Participant selection was carried out through direct clinical examination and medical records at the Odonto Maxillofacial Hospital in Ho Chi Minh City, Vietnam. Written informed consent was obtained from all subjects or their legal guardians after the objectives and procedures of the study were clearly explained.

Peripheral venous blood samples were collected using dried blood spot (DBS) cards, which were subsequently preserved at the World Cleft Gene Bank, Aichi Gakuin University, Nagoya, Japan. All study procedures were performed in compliance with the principles outlined in the Declaration of Helsinki, and the research protocol received ethical approval from the Aichi Gakuin University Ethics Committee (Approval No. 689) on 4 December 2023.

### 2.2. Single-Nucleotide Polymorphism Selection

This study focused on investigating *WNT3* rs3809857 and *NOG* rs227731, based on findings from previous genome-wide association studies (GWAS) and meta-analysis [17,19]. In addition, we utilized the dbSNP database and the 1000 Genomes Browser to identify and confirm previously reported associations [20]. Both single-nucleotide polymorphisms (SNPs) have been proposed as potential genetic markers involved in the etiology of non-syndromic orofacial clefts.

### 2.3. DNA Extraction and Genotyping

Genomic DNA was extracted from dried blood samples using the Nucleospin Tissue Kit (MACHEREY-NAGEL, Düren, Germany). The quality and concentration of the extracted DNA were assessed using spectrophotometric analysis.

For genotyping, the TaqMan SNP Genotyping Assay (Applied Biosystems, Foster City, CA, USA) was employed, using the StepOnePlus Real-Time PCR System (Thermo Fisher Scientific, Waltham, MA, USA). The genotyping procedure was performed following the manufacturer’s instructions, utilizing the dried DNA method.

### 2.4. Statistical Analysis

Hardy-Weinberg Equilibrium (HWE) was tested for 2 SNPs among healthy controls using Pearson’s chi-square statistics. Comparison of the genotypic (aa or Aa vs. AA), dominant (AA vs. Aa+aa), recessive models (aa vs. Aa+AA), and allele frequencies (a vs. A) between case and control groups was performed using the chi-squared test or Fisher’s exact test. Odds ratios (ORs) and 95% confidence intervals (CIs) were also calculated. Two SNPs were tested for association with 3 NSOFC subtypes using the Cochran–Armitage trend test. A Bonferroni correction was applied to multiple tests to determine the critical thresholds of statistical significance. In this study, we used Bonferroni correction for 12 tests (2 SNPs × 3 phenotypes × 2 genders) with α = 0.05/12 = 0.00417. A P-value of less than 0.00417 was considered statistically significant after applying the Bonferroni correction.

A priori power analysis was performed using G*Power (version 3.1.9.7) to determine the necessary sample size. The analysis indicated that a minimum of 349 participants is required to achieve 80% power (1−β) for detecting a moderate effect size (0.15), at a significance level (α) of 0.05. Exceeding this requirement, our study recruited 400 participants for each subtype comparison (160 cases, 240 controls), including NSCLP vs. control, NSCLO vs. control, and NSCPO vs. control, ensuring statistical power for every subgroup analysis.

## 3. Results

None of the HWE tests with two SNPs showed a significant deviation in healthy individuals with HWE *p* > 0.05, suggesting good homogeneity within the study subjects. Minor allele frequencies (MAFs) were all above 20% (Table 1).

### 3.1. WNT3 rs3809857

In males NSCLP group, this study showed the significant differences between case and control in allelic, genotypic and dominant model with the OR was 0.61(95%CI: 0.40–0.95, *p* = 0.0293), 0.53 (95%CI: 0.28–1.00, *p* = 0.0496), 0.53(95%CI: 0.30–0.94, *p* = 0.0282), respectively. These findings revealed the decreasing risk of NSCLP of allele T, genotype GT, and TT, though these associations did not remain significant after applying Bonferroni correction for multiple testing (Table 2). Additionally, the genotypic prevalence illustrated the significance of the Cochran-Armitage trend test with *p* = 0.049. Conversely, in the female cluster, rs3809857 was not significantly associated with NSCLP across all model tests.

Significant differences were found in the males NSCLO group compared with controls in genotype TT, allele T, and the recessive model. Especially, this study identified a strong association in the recessive model even after Bonferroni correction with OR = 0.18 (95%CI: 0.05–0.64, *p* = 0.0033). The genotypic prevalence illustrated the significance of the Cochran-Armitage trend test with *p* = 0.0184. Conversely, there were no significant findings in the female NSCLO group (Table 3).

No influence on the risk for NSCPO was observed for both male and female groups (Table 4).

### 3.2. NOG rs227731

In NSCLP, there was no evidence for association in both male and female groups (Table 2). The homozygous genotype (CC) distribution increased NSCLO and NSCPO susceptibility in the female group, with the *p* value 0.0377 and 0.0257, respectively. Additionally, the recessive genetic model revealed a significant difference between female NSCPO and the control with *p* = 0.0145, though these associations did not remain significant after applying Bonferroni adjustment. However, the significant findings were not found in the male NSCLO and NSCPO.

### 3.3. Gene-Gene-Sex Interaction

To test whether a second risk or protective gene would further increase or decrease the disease prevalence, all significant groups were tested for the gene-gene-sex interaction. The results showed no significant findings in all orofacial cleft subtypes with *p* > 0.05 (Table 5).

## 4. Discussion

The Minor Allele Frequency for rs3809857 in our study cohort was 0.3333, a value higher than that reported from the 1000 Genomes Project for general Asian (0.27) and global (0.31) populations [20]. This difference can be explained by our recruitment strategy and population-specific genetics. Our study utilized a hospital-based sample with a balanced 1:1 male-to-female ratio, which differs from natural population distributions and may introduce sampling bias. Furthermore, this discrepancy likely reflects the unique genetic profile of our participants, as significant regional genetic diversity is known to exist within the Vietnamese population, particularly between northern and southern regions and among different ethnic groups.

Non-syndromic orofacial clefts are a multifactorial condition influenced by both genetic predispositions and environmental exposures [21]. Among the genetic contributors, the WNT signaling pathway plays a pivotal role in embryogenesis, particularly in regulating key cellular processes involved in primary lip and palate formation, as well as secondary palate development [9,22]. *WNT3*, a member of this pathway, exhibits pleiotropic functions during embryonic development, as demonstrated in murine models and supported by evidence from human and mouse genetic studies [15,19]. The SNP rs3809857 in *WNT3* has been investigated in relation to NSCL/P, revealing complex and sometimes inconsistent associations across populations [19]. As rs3809857 is located within an intron of the *WNT3* gene, it does not directly alter the protein sequence. However, intronic SNPs can still influence gene expression and function through various mechanisms, such as affecting transcription factor binding, splicing, or the stability of non-coding RNA, thereby influencing the risk of NSCL/P [19]. In the current case-control study, the *WNT3* rs3809857 TT genotype was significantly associated with a reduced risk of NSCLO in males, suggesting that individuals with this genotype had approximately a five-fold decreased risk. Similar protective factors were reported in a Northeast Chinese population, where both case-control (236 cases, 400 controls) and family-based (128 trios) analyses identified the T allele as protective [23]. Additionally, an Iranian study (113 cases, 220 controls) found a lower frequency of the GT genotype among cases [24]. In contrast, a recent study in a Polish population (209 cases, 418 controls) did not observe a statistically significant association between rs3809857 and NSCL/P, highlighting potential ethnic and genetic heterogeneity in NSOFCs susceptibility [25].

Epidemiological studies have consistently shown that males exhibit a higher prevalence of cleft lip with or without palate compared to females [5]. The present findings emphasize the significant role of *WNT3* rs3809857 in craniofacial development and, more specifically, suggest sex-specific genetic susceptibility to NSCLO. Explanations for these findings have been elusive, but this sex disparity may be driven by several biological mechanisms, such as hormonal influences during key periods of craniofacial morphogenesis that could interact with *WNT3* function or expression [26]. Additionally, a previous study examining female discordant twin and sibling pairs with CL/P through X-chromosome inactivation patterns suggested a potential role for epigenetic regulation in CL/P aetiology [27]. Although there was no available evidence for an interaction between WNT3 and sex loci before. As a potential hypothesis, sex-specific susceptibility may be influenced by genetic factors located on the X or Y chromosomes, potentially conferring risk in males but not in females. Another potential explanation involves sex-dependent liability thresholds, wherein identical risk alleles exert equal effects in both sexes, but females may require a greater cumulative burden of risk alleles or more highly penetrant mutations to manifest the phenotype [7]. Therefore, the present study should be interpreted as hypothesis-generating, as the observed gene-by-sex (G × S) interactions. The observed male-specific protective effect of the rs3809857 variant highlights the need for further research to clarify the molecular basis of this sexual dimorphism in NSCL/P susceptibility.

Noggin is a well-characterized extracellular antagonist of specific members of the Transforming Growth Factor-beta (TGF-β) superfamily, particularly Bone Morphogenetic Proteins (BMPs), which are essential for maintaining palatal epithelial integrity and normal palatogenesis [16]. Experimental studies in Nog-knockout mice have demonstrated severe craniofacial malformations, including cleft palate and mandibular overgrowth, underscoring the critical role of noggin in craniofacial development [16]. In the present study, the *NOG* rs227731 variant was found to be potentially associated with female NSCLO and NSCPO. Although these tests were not significant after applying the Bonferroni correction, the results may suggest a potential direction for future studies to clarify these associations. This SNP was initially identified in GWAS as a strong susceptibility marker, particularly in European populations [17]. Other studies reported significant associations between rs227731 and NSCLP as a risk factor in Polish (dominant model test with OR = 1.73) [28]. Moreover, the findings from a meta-analysis imply that the rs227731 variant could contribute to a heightened susceptibility to NSCL/P among Caucasians, with odds ratios ranging from approximately 1.4 to 2.0, whereas no meaningful association was observed within the Chinese population [18].

The investigation of genetic factors underlying NSOFCs in the Vietnamese population remains limited. Previous studies from our laboratory have identified associations between NSOFCs and genes such as *MSX1*, *IRF6*, and *TFAP2A* [29,30,31]. Additionally, research by Duy et al. revealed a gene–sex interaction involving NSCPO in female subjects [8]. The present study examined all subtypes of NSOFCs and identified a gene–sex association between *WNT3* and NSCLO. Furthermore, weak or moderate associations from the present study that did not reach statistical significance after Bonferroni correction should still be considered as potential findings that suggest further investigation.

This study still contains certain limitations, including a relatively small sample size and a single population, which may restrict the generalizability of the findings. Future studies should aim to replicate the observed sex-specific protective association in larger and more ethnically diverse male populations. The generalizability of our findings is constrained by the hospital-based recruitment, case-control approach with balanced recruitment for each cleft subtype (NSCLO, NSCLP, and NSCPO). Moreover, significant phenotypic heterogeneity within each cleft subtype may obscure genetic associations specific to more granular clinical features, such as cleft severity or laterality. Additionally, our results may be affected by unmeasured confounding variables, such as specific maternal environmental exposures that can be improved in further study with gene-environment interaction. Although the Bonferroni correction is a reliable tool for avoiding Type I errors, it is sometimes too strict and may underestimate the weak or moderate genetic associations. Therefore, we sometimes decided not to apply this adjustment.

## 5. Conclusions

In conclusion, this study identifies a significant association between the *WNT3* rs3809857 polymorphism and a reduced risk of NSCLO in male Vietnamese. These findings support the critical role of *WNT3* in craniofacial development and highlight the potential influence of sex as a modifying factor in the genetic susceptibility to non-syndromic orofacial clefts. Further studies are needed to clarify the interaction between *NOG* and the sex factor.

## Figures and Tables

**Table 1:** Minor allele frequency and Hardy–Weinberg equilibrium test.

Genes	SNPs	Alleles	HWEp	MAF			
				Control	NSCLP	NSCLO	NSCPO
*WNT3*	rs3809857	G>T	0.1213	0.3333	0.3031	0.2781	0.2750
*NOG*	rs227731	A>C	0.2636	0.3000	0.3000	0.3689	0.2938

Abbreviation: SNPs, single-nucleotide polymorphisms; HWEp, Hardy–Weinberg equilibrium *p*-value; MAF, minor allele frequency.

**Table 2:** Association of *WNT3* rs3809857 and *NOG* rs227731 with NSCLP in male and female clusters.

SNPs	Genotype/ Allele	Case/Control (Males)	Case/ Control (Females)	OR_male_ 95%CI/ *p*-Value **	OR_female_ 95%CI/ *p*-Value **	P_trend_ Males	P_trend_ Female
*WNT3* Rs3809857	GG	48/53	35/59	1	1	**0.049**	0.3617
	GT	22/46	35/50	**0.53 (0.28–1.00)/0.0496**	1.18 (0.65–2.15)/0.5894		
	TT	10/21	10/11	0.53 (0.23–1.23)/0.1340	1.53 (0.59–3.97)/0.3780		
	G	118/152	105/168	1	1		
	T	42/88	55/72	**0.61 (0.40–0.95)/0.0293**	1.22 (0.80–1.87)/0.3571		
	Dominant	GG/GT+TT		**0.53 (0.30–0.94)/0.0282**	1.24 (0.70–2.20)/0.4521		
	Recessive	TT/GG+GT		0.67 (0.30–1.52)/0.3385	1.42 (0.57–3.51)/0.4512		
*NOG* rs227731	AA	33/56	38/53	1	1	0.9239	0.9179
	AC	44/54	38/64	1.38 (0.77–2.48)/0.2779	0.83 (0.46–1.48)/0.5227		
	CC	3/10	4/3	0.51 (0.13–1.98)/0.3732 *	1.86 (0.39–8.80)/0.4575*		
	A	110/166	114/170	1	1		
	C	50/74	46/70	1.02 (0.66–1.57)/0.9297	0.98 (0.63–1.52)/0.9283		
	Dominant	AA/AC+CC		1.25 (0.70–2.21)/0.4502	0.87 (0.50–1.54)/0.6428		
	Recessive	CC/AA+AC		0.43 (0.11–1.61)/0.2503 *	2.05 (0.45–9.43)/0.4408 *		

Significant results are highlighted in bold font (not corrected for multiple tests); * Fisher’s exact test; ** Chi-square analysis; Ptrend, Cochran-Armitage trend test.

**Table 3:** Association of *WNT3* rs3809857 and *NOG* rs227731 with NSCLO in male and female clusters.

SNPs	Genotype/ Allele	Case/Control (Males)	Case/ Control (Females)	OR_male_ 95%CI/ *p*-Value **	OR_female_ 95%CI/ *p*-Value **	P_trend_ Males	P_trend_ Female
*WNT3* Rs3809857	GG	43/53	42/59	1	1	**0.0184**	0.8983
	GT	34/46	27/50	0.91 (0.50–1.66)/0.7602	0.76 (0.41–1.40)/0.3765		
	TT	3/21	11/11	**0.18** (**0.05–0.63**)/0.0042 *	1.40 (0.56–3.54)/0.4701		
	G	120/152	111/168	1	1		
	T	40/88	49/72	**0.58** (**0.37–0.90**)**/0.0143**	1.03 (0.67–1.59)/0.8939		
	Dominant	GG/GT+TT		0.68 (0.39–1.20)/0.1839	0.88 (0.50–1.54)/0.6442		
	Recessive	TT/GG+GT		**0.18** (**0.05–0.64**)/0.0033 *^€^	1.58 (0.65–3.84)/0.3101		
*NOG* rs227731	AA	30/56	27/53	1	1	0.2379	**0.0436**
	AC	42/54	46/64	1.45 (0.80–2.64)/0.2220	1.41 (0.78–2.57)/0.2590		
	CC	8/10	7/3	1.49 (0.53–4.18)/0.4437	**4.58** (**1.10–19.13**)/0.0377 *		
	A	102/166	100/170	1	1		
	C	58/74	60/70	1.28 (0.84–1.95)/0.2590	1.46 (0.95–2.23)/0.0813		
	Dominant	AA/AC+CC		0.58 (0.33–1.03)/0.0645	1.55 (0.86–2.79)/0.1407		
	Recessive	CC/AA+AC		1.22 (0.46–3.24)/0.6866	3.74 (0.94–14.9)/0.0927 *		

Significant results are highlighted in bold font (not corrected for multiple tests); * Fisher’s exact test; ** Chi-square analysis; ^€^ Significant with Bonferroni correction for multiple tests; Ptrend, Cochran-Armitage trend test.

**Table 4:** Association of *WNT3* rs3809857 and *NOG* rs227731 with NSCPO in male and female clusters.

SNPs	Genotype/ Allele	Case/Control (Males)	Case/ Control (Females)	OR_male_ 95%CI/ *p*-Value **	OR_female_ 95%CI/ *p*-Value **	P_trend_ Males	P_trend_ Female
*WNT3* Rs3809857	GG	37/53	49/59	1	1	**0.049**	0.3617
	GT	35/46	25/50	1.09 (0.59–2.00)/0.7814	0.60 (0.33–1.11)/0.1027		
	TT	8/21	6/11	0.55 (0.22–1.36)/0.1915	0.66 (0.23–1.90)/0.4366		
	G	109/152	123/168	1	1		
	T	51/88	37/72	0.32 (0.53–1.23)/0.3242	0.70 (0.44–1.11)/0.1303		
	Dominant	GG/GT+TT		0.92 (0.52–1.62)/0.7717	0.61 (0.34–1.09)/0.0930		
	Recessive	TT/GG+GT		0.52 (0.22–1.25)/0.1401	0.80 (0.28–2.27)/0.0930		
*NOG* rs227731	AA	48/56	32/53	1	1	0.9239	0.9179
	AC	27/54	39/64	0.58 (0.32–1.06)/0.0781	1.01 (0.56–1.83)/0.9756		
	CC	5/10	9/3	0.58 (0.19–1.83)/0.4141 *	**4.97** (**1.25–19.72**)/0.0257 *		
	A	123/166	103/170	1	1		
	C	37/74	57/70	0.67 (0.43–1.07)/0.0917	1.34 (0.88–2.06)/0.1740		
	Dominant	AA/AC+CC		0.58 (0.33–1.03)/0.0645	1.19 (0.67–2.11)/0.5592		
	Recessive	CC/AA+AC		0.73(0.24–2.23)/0.7852 *	4.94(1.30–18.87)/0.0145 *		

Significant results are highlighted in bold font (not corrected for multiple tests); * Fisher’s exact test; ** Chi-square analysis; Ptrend, Cochran-Armitage trend test.

**Table 5:** Gene-Gene-Sex Interaction in Male and Female Clusters.

Genders	Phenotypes	Both Genes	*WNT3*	*p*-Value **	OR (95%CI)	*NOG*	*p*-Value **	OR (95%CI)
Male	NSCLP	17	15	0.7366	0.86 (0.37–2.02)	4	0.1133 *	2.91 (0.88–9.64)
	NSCLO	25	12	0.2783	1.59 (0.69–3.69)	25	0.3178	0.68 (0.32–1.44)
	Control	38	29	-	-	26	-	-
Female	NSCLO	24	14	0.9312	1.04 (0.45–2.40)	29	0.2133	0.63 (0.31–1.30)
	NSCPO	19	12	0.9252	0.96 (0.39–2.33)	29	0.0700	0.5 (0.24–1.06)
	Control	38	23	-	-	29	-	-

* Fisher’s exact test; ** Chi-square analysis.

## Data Availability

The original contributions presented in this study are included in the article. Further inquiries can be directed to the corresponding authors.

## References

[1] Mossey P.A., Little J., Munger R.G., Dixon M.J., Shaw W.C. (2009). Cleft lip and palate. Lancet.

[2] Dixon M.J., Marazita M.L., Beaty T.H., Murray J.C. (2011). Cleft lip and palate: Understanding genetic and environmental influences. Nat. Rev. Genet..

[3] Natsume N., Kawai T., Le H. (1998). In Vietnam, many congenital anomalies are believed to result from the scattering of defoliants, including dioxin. Cleft Palate Craniofac. J..

[4] De Cuyper E., Dochy F., De Leenheer E., Van Hoecke H. (2019). The impact of cleft lip and/or palate on parental quality of life: A pilot study. Int. J. Pediatr. Otorhinolaryngol..

[5] Marazita M.L. (2012). The Evolution of Human Genetic Studies of Cleft Lip and Cleft Palate. Annu. Rev. Genom. Hum. Genet..

[6] Awotoye W., Comnick C., Pendleton C., Zeng E., Alade A., Mossey P.A., Gowans L.J.J., Eshete M.A., Adeyemo W.L., Naicker T. (2021). Genome-wide Gene-by-Sex Interaction Studies Identify Novel Nonsyndromic Orofacial Clefts Risk Locus. J. Dent. Res..

[7] Carlson J.C., Nidey N.L., Butali A., Buxo C.J., Christensen K., Deleyiannis F.W.D., Hecht J.T., Field L.L., Moreno-Uribe L.M., Orioli I.M. (2018). Genome-wide interaction studies identify sex-specific risk alleles for nonsyndromic orofacial clefts. Genet. Epidemiol..

[8] Tran D.L., Imura H., Mori A., Suzuki S., Niimi T., Ono M., Sakuma C., Nakahara S., Nguyen T.T.H., Pham P.T. (2018). Association of MEOX2 polymorphism with nonsyndromic cleft palate only in a Vietnamese population. Congenit. Anom..

[9] Mani P., Jarrell A., Myers J., Atit R. (2010). Visualizing canonical Wnt signaling during mouse craniofacial development. Dev. Dynam..

[10] Vijayan V., Ummer R., Weber R., Silva R., Letra A. (2018). Association of WNT Pathway Genes with Nonsyndromic Cleft Lip with or Without Cleft Palate. Cleft Palate Craniofac. J..

[11] Marchini M., Hu D., Lo Vercio L., Young N.M., Forkert N.D., Hallgrímsson B., Marcucio R. (2021). Wnt Signaling Drives Correlated Changes in Facial Morphology and Brain Shape. Front. Cell Dev. Biol..

[12] Ferretti E., Li B., Zewdu R., Wells V., Hebert Jean M., Karner C., Anderson Matthew J., Williams T., Dixon J., Dixon Michael J. (2011). A Conserved Pbx-Wnt-p63-Irf6 Regulatory Module Controls Face Morphogenesis by Promoting Epithelial Apoptosis. Dev. Cell..

[13] Maili L., Letra A., Silva R., Buchanan E.P., Mulliken J.B., Greives M.R., Teichgraeber J.F., Blackwell S.J., Ummer R., Weber R. (2020). PBX-WNT-P63-IRF6 pathway in nonsyndromic cleft lip and palate. Birth. Defects. Res..

[14] Juriloff D.M., Harris M.J. (2008). Mouse genetic models of cleft lip with or without cleft palate. Birth. Defects. Res. A Clin. Mol. Teratol..

[15] Lan Y., Ryan R.C., Zhang Z., Bullard S.A., Bush J.O., Maltby K.M., Lidral A.C., Jiang R. (2006). Expression of Wnt9b and activation of canonical Wnt signaling during midfacial morphogenesis in mice. Dev. Dyn..

[16] He F., Xiong W., Wang Y., Matsui M., Yu X., Chai Y., Klingensmith J., Chen Y. (2010). Modulation of BMP signaling by Noggin is required for the maintenance of palatal epithelial integrity during palatogenesis. Dev. Biol..

[17] Mangold E., Ludwig K.U., Birnbaum S., Baluardo C., Ferrian M., Herms S., Reutter H., de Assis N.A., Chawa T.A., Mattheisen M. (2010). Genome-wide association study identifies two susceptibility loci for nonsyndromic cleft lip with or without cleft palate. Nat. Genet..

[18] Wang F., Jiang Y., Yang S., Liu Q., Lin J., Zhang H. (2017). Associations between the NOGGIN rs227731 polymorphism and NSCL/P risk may be associated with ethnicities: A meta-analysis. Birth. Defects. Res..

[19] Wang B.Q., Gao S.T., Chen K., Xu Z.Q., Sun J.M., Xia Y., Lv Z.T. (2018). Association of the WNT3 polymorphisms and non-syndromic cleft lip with or without cleft palate: Evidence from a meta-analysis. Biosci. Rep..

[20] Auton A., Brooks L.D., Durbin R.M., Garrison E.P., Kang H.M., Korbel J.O., Marchini J.L., McCarthy S., McVean G.A., Abecasis G.R. (2015). A global reference for human genetic variation. Nature.

[21] Carinci F., Scapoli L., Palmieri A., Zollino I., Pezzetti F. (2007). Human genetic factors in nonsyndromic cleft lip and palate: An update. Int. J. Pediatr. Otorhinolaryngol..

[22] Menezes R., Letra A., Kim A.H., Küchler E.C., Day A., Tannure P.N., da Motta L.G., Paiva K.B.S., Granjeiro J.M., Vieira A.R. (2010). Studies with Wnt genes and nonsyndromic cleft lip and palate. Birth. Defects. Res. A Clin. Mol. Teratol..

[23] Lu Y.P., Han W.T., Liu Q., Li J.X., Li Z.J., Jiang M., Xu W. (2015). Variations in WNT3 gene are associated with incidence of non-syndromic cleft lip with or without cleft palate in a northeast Chinese population. Genet. Mol. Res..

[24] Farrokhi Karibozorg H., Masoudian N., Saliminejad K., Ebadifar A., Kamali K., Khorram Khorshid H.R. (2018). Association of the WNT3 Variations and the Risk of Non-Syndromic Cleft Lip and Palate in a Population of Iranian Infants. Avicenna. J. Med. Biotechnol..

[25] Zawiślak A., Woźniak K., Tartaglia G., Agirre X., Gupta S., Kawala B., Znamirowska-Bajowska A., Grocholewicz K., Prosper F., Lubiński J. (2024). Single-Nucleotide Polymorphisms in WNT Genes in Patients with Non-Syndromic Orofacial Clefts in a Polish Population. Diagnostics.

[26] Wu W., Gu S., Sun C., He W., Xie X., Li X., Ye W., Qin C., Chen Y., Xiao J. (2015). Altered FGF Signaling Pathways Impair Cell Proliferation and Elevation of Palate Shelves. PLoS ONE.

[27] Kimani J.W., Shi M., Daack-Hirsch S., Christensen K., Moretti-Ferreira D., Marazita M.L., Field L.L., Canady J.W., Murray J.C. (2007). X-chromosome inactivation patterns in monozygotic twins and sib pairs discordant for nonsyndromic cleft lip and/or palate. Am. J. Med. Genet. Part A.

[28] Mostowska A., Hozyasz K.K., Wojcicka K., Biedziak B., Jagodzinski P.P. (2012). Polymorphic variants at 10q25.3 and 17q22 loci and the risk of non-syndromic cleft lip and palate in the polish population. Birth. Defects. Res. A Clin. Mol. Teratol..

[29] Suzuki Y., Jezewski P.A., Machida J., Watanabe Y., Shi M., Cooper M.E., Viet L.T., Tin N.T.D., Hai H., Natsume N. (2004). In a Vietnamese population, MSX1 variants contribute to cleft lip and palate. Genet. Med..

[30] Nguyen D.M., Suzuki S., Imura H., Niimi T., Furukawa H., Ta T.-V., Tong S.M., Nguyen T.T., Pham L.N.G., Tran D.L. (2021). Family based and case–control designs reveal an association of TFAP2A in nonsyndromic cleft lip only among Vietnamese population. Mol. Genet. Genomic Med..

[31] Pham L.N.G., Niimi T., Suzuki S., Nguyen M.D., Nguyen L.C.H., Nguyen T.D., Hoang K.A., Nguyen D.M., Sakuma C., Hayakawa T. (2023). Association between IRF6, TP63, GREM1 Gene Polymorphisms and Non-Syndromic Orofacial Cleft Phenotypes in Vietnamese Population: A Case-Control and Family-Based Study. Genes.

